# A Retrospective Analysis of Guanfacine for the Pharmacological Management of Delirium

**DOI:** 10.7759/cureus.33393

**Published:** 2023-01-05

**Authors:** Shixie Jiang, Michael Hernandez, Heather Burke, Benjamin Spurling, Richard Czuma, Rojan Varghese, Alexis Cohen, Kimberly Hartney, Gregory Sullivan, F. Andrew Kozel, Jose R Maldonado

**Affiliations:** 1 Psychiatry, Stanford University School of Medicine, Stanford, USA; 2 Psychiatry, University of South Florida, Tampa, USA; 3 Psychiatry, Florida State University, Tallahassee, USA

**Keywords:** opioid, alpha-2 agonist, agitation, hyperactive delirium, mixed delirium, guanfacine, delirium

## Abstract

Background

Delirium is a syndrome of acute brain failure that represents a change from an individual's baseline cognitive functioning characterized by deficits in attention and multiple aspects of cognition that fluctuate in severity over time. The symptomatic management of delirium's behavioral manifestations remains difficult. The alpha-2 agonists, dexmedetomidine and clonidine, are efficacious, but their potential cardiovascular adverse effects limit their utilization. Guanfacine is an oral alpha-2 agonist with a lower potential for such adverse outcomes; however, its use in delirium has not been studied.

Methods

A retrospective descriptive analysis of guanfacine for managing hyperactive or mixed delirium at Tampa General Hospital from January 2020 to October 2020 was conducted. The primary outcome was the time reduction in acute sedative administration. Secondary outcomes included renewed participation in physical therapy or occupational therapy (PT/OT), decreased opioid use, and an incidence of cardiovascular adverse effects.

Results

One hundred forty-nine patients were identified as having received guanfacine for managing delirium during the study period. All experienced a reduction in acute sedative use after the initiation of guanfacine. In 93 patients receiving PT/OT and no longer participating due to behavioral agitation, 74% had a documented renewal of services within four days. Of 112 patients on opioids, 70% experienced a 25% reduction in opioid administration within four days. No patients experienced consecutive episodes of hypotension that required a change in their clinical care. Two patients experienced a single episode of consecutive bradycardia that led to the discontinuation of guanfacine.

Conclusions

Based on our retrospective study, guanfacine is a well-tolerated medication for the management of delirium. Even in medically and critically ill patients, cardiovascular adverse events were rare with guanfacine. Patients treated with guanfacine experienced decreased acute sedative use for behavioral agitation. Additionally, patients treated with guanfacine received fewer opioids and were better able to participate in PT/OT. Future studies with prospective, randomized, placebo-controlled designs are warranted to evaluate this promising intervention for delirium further.

## Introduction

Delirium is a syndrome of acute brain failure that represents a change from an individual's baseline cognitive functioning characterized by deficits in attention, awareness, and multiple aspects of cognition that fluctuate in severity over time. It is the most common neuropsychiatric disorder observed in hospitalized patients, affecting 10% to 60% of medical inpatients with a general medical disorder and up to 82% of mechanically-ventilated patients in the intensive care unit (ICU) [1]. Among the classic delirium phenotypes, the hyperactive and mixed subtypes remain the more challenging to manage, given the resultant episodes of behavioral agitation that significantly disrupt patient care [2,3]. As such, the optimal pharmacologic treatment of delirious agitation remains a prime research endeavor, as ameliorating these behavioral events leads to improved outcomes, decreased hospitalization costs, and a higher quality of care [4]. 

Antipsychotics have historically been used for the management of delirium's behavioral manifestations. In an international survey of 1521 intensivists, 65% reported that they manage delirium in the ICU with haloperidol, and 53% reported using a second-generation antipsychotic [5]. The dose of antipsychotic required to achieve the desired levels of sedation can be, at times, excessive or even extraordinary [6-8]. Many studies on the use of antipsychotics in delirium are anchored on the reported finding of excessive dopaminergic tone in delirium models [9-13]. These drugs either directly or indirectly antagonize synaptic dopamine transmission. They are thought to putatively alleviate hippocampal dysfunction (e.g., memory impairments) and other symptoms, including aggression, violence, anxiety, and psychotic phenomena, by this mechanism [1]. However, the level of dopamine activity in delirium is highly variable and even decreased in some types of delirium depending on the individual etiology (e.g., hepatic encephalopathy, sleep deprivation, trauma, and post-operative settings) [14]. There may be many reasons for the reported mixed outcomes in clinical trials. As it pertains to cases of hyperactive/mixed-type delirium, more recent data also suggests that most symptoms are likely mediated by excess activity of glutamate or norepinephrine [14].

Furthermore, antipsychotics are not without potentially serious adverse effects, including cardiac (e.g., QT prolongation, hypotension, and arrhythmias), cerebrovascular events, central nervous system events (e.g., somnolence), anticholinergic effects, and overall increased mortality [15,16]. This is especially important as many of these agents, and their related side effects might contribute to the onset or perpetuation of delirium. Despite such controversy, antipsychotics continue to be routinely administered as our pharmacologic armamentarium for delirious agitation remains limited.

Recently, research into alpha-2 agonists, such as dexmedetomidine and clonidine, has been pursued with great interest due to their reported efficacy in preventing and managing delirium [1,16,17]. These agents exert their therapeutic effects by modulating alpha-2A, 2B, and 2C receptors and subsequently inhibit the presynaptic release of norepinephrine (NE) [8,9]. Delirium models have supported an excess of NE, which propagates neuronal damage and cell death, thus worsening symptomatology [14,18]. Additionally, these alpha-2 agonists may inhibit glutamate release via the suppression of voltage-dependent calcium channels and mitogen-activated protein kinase [19]. This leads to further mitigation of excitatory neurotransmitter activity and its deleterious effects. Clinically, this combined mechanism of action is likely responsible for the sedative, anxiolytic, anti-nociceptive, and sleep-promoting effects of these medications [20-22]. Dexmedetomidine, in particular, has repeatedly demonstrated robust preventative and treatment capabilities in multiple larger clinical trials [23,24]. Unfortunately, the use of these drugs is limited due to the incidence of significant cardiovascular adverse effects, including hypotension and bradycardia. Moreover, dexmedetomidine requires an ICU level of monitoring and is not available outside such a setting [25].

Guanfacine is another alpha-2 agonist typically used to treat attention deficit/hyperactivity disorder (ADHD). It possesses a much higher alpha-2 receptor selectivity than dexmedetomidine and clonidine. It is reportedly 25 times more selective than clonidine and 1.65 times more selective than dexmedetomidine [26-28]. This significantly higher alpha-2 selectivity may lead to diminished cardiovascular adverse effects, as increased alpha-2 selectivity coincides with decreased selectivity for the hypotension-inducing imidazole receptor [29]. Given this difference in the mechanisms of action, Maldonado previously proposed the advantage of guanfacine in managing delirium [1]. Since then, only a small case series has published positive results, with no formal studies conducted [30]. Our group presented preliminary results of guanfacine's potential efficacy at the Academy of Consultation-Liaison Psychiatry Annual Conference in November 2022 [31]. 

We have conducted a retrospective analysis of guanfacine for managing delirium in our hospital's ICU and general wards. The primary outcome was the time to 25% reduction in acute sedative (rescue sedation) administration after initiation of guanfacine. Our secondary outcomes included renewed participation in physical therapy or occupational therapy (PT/OT) after initiation of guanfacine, opioid use after initiation of guanfacine, and the incidence of cardiovascular adverse effects after administration of guanfacine.

## Materials and methods

For this retrospective descriptive analysis, data were collected from an Epic electronic medical database via Epic SlicerDicer software at the Tampa General Hospital in Tampa, FL, USA. Records from January 2020 to October 2020 were retrieved for this study. Only patients diagnosed with delirium and for whom guanfacine (Tenex) was specifically administered for symptoms of delirium were included. The diagnosis of delirium was initially recorded by either the psychiatric consultant who evaluated the patient or the primary service team physician(s). Confirmation of the diagnosis of delirium and its subtyping (mixed or hyperactive) was established by the consulting psychiatrist based on the Diagnostic and Statistical Manual of Mental Disorders, 5th Edition [DSM-5][32] and Liptzin-Levkoff criteria [33], respectively, for all patients included in this analysis. Subjects with a diagnosis of hypoactive delirium were excluded from our sample.

Electronic medical records were reviewed for demographic and clinical characteristics, diagnoses (International Classification of Diseases, 10th Revision, Clinical Modification [ICD-10-CM]) [34], hospital service type (medical or surgical), sedative agents and their respective dosages, guanfacine initiation day and dosing, time (in hours) until a 25% reduction in acute sedative use (rescue sedation) for agitation, dose of each acute sedative agent prior to start of guanfacine (24 hour average), renewal of previously prescribed physical therapy and/or occupational therapy participation within four days of guanfacine initiation (if not actively receiving), dose of opioid agents prior to start of guanfacine (24 hour average), 25% reduction in opioid administration within four days of guanfacine initiation, guanfacine use during QTc prolongation (i.e., Fridericia criteria ≥ 500 msec) [35], or adverse cardiovascular event(s), including clinically significant bradycardia (defined as two or more consecutive measurements of HR<50 beats per minute or hypotension (defined as two or more consecutive measurements of SBP<90 mmHg and/or DBP<60 mmHg), either requiring a clinical intervention or change in plan of care.

A 25% reduction in acute sedative use was chosen as the primary outcome. This parameter can be used as a marker for a reduction in basal agitation in the absence of prospective delirium severity scale scores. The initial dosing and titration of guanfacine were based on individual clinical judgment and expertise. Continuous data are presented as means ± standard deviations (SD), and categorical data as percentages.

This study was approved by the University of South Florida Institutional Review Board as an exempted retrospective study and determined not to require informed consent.

## Results

In total, 149 patients were identified as meeting the inclusion criteria of having received guanfacine for managing delirium during the defined study period (Table 1). The average age (±SD) of these patients was 58.12 ± 16.67 years, range of 19 to 89 years, with 67.11% males. The mean weight was 80.82 ± 21.87 kilograms. The ethnicities of these individuals included Caucasian (63.76%), African-American (21.48%), Hispanic (7.38%), Asian (6.71%), and Unknown (0.67%). Patients were admitted to the hospital for a wide variety of reasons: 23 patients (15.44%) were admitted for a cardiovascular diagnosis; 21 (14.09%) for a respiratory diagnosis; 20 (13.42%) for a neurologic diagnosis; 18 (12.08%) for a hepatic diagnosis; 16 (10.74%) for sepsis or infectious disease; 15 (10.07%) for a renal diagnosis; 13 (8.72%) for gastrointestinal diagnosis; and 23 (15.44%) for other/multi-component. A total of 105 patients (70.47%) were admitted under a medical service, and 44 (29.53%) were admitted under a surgical service. Relevant characteristics of their co-morbidities can also be found in Table 1. Critically ill patients comprised the majority of our sample, with 95 (63.76%) in the ICU at the time of guanfacine initiation and 54 (36.24%) in the hospital's general wards. Most of the sample (n=128, 85.91%) was diagnosed with hyperactive delirium, with the remaining patients (n=21, 14.09%) diagnosed with mixed delirium.

**Table 1:** Demographic data of patients with delirium receiving guanfacine (n=149).

Characteristics	Result
Age (mean ± SD and range in years)	58.12 ± 16.67; range of 19 to 89 years
Gender (n, %)	
Male	100 (67.11%)
Female	49 (32.89%)
Ethnicity (n, %)	
Caucasian	95 (63.76%)
African-American	32 (21.48%)
Hispanic	11 (7.38%)
Asian	10 (6.71%)
Unknown	1 (0.67%)
Weight (mean ± SD, kg)	80.82 ± 21.87
Service Type (n, %)	
Medical	105 (70.47%)
Surgical	44 (29.53%)
Admission diagnosis (n, %)	
Cardiovascular	23 (15.44%)
Other/multi-component	23 (15.44%)
Respiratory	21 (14.09%)
Neurologic	20 (13.42%)
Hepatic	18 (12.08%)
Sepsis/infectious disease	16 (10.74%)
Renal	15 (10.07%)
Gastrointestinal	13 (8.72%)
Admission setting (n, %)	
Intensive care unit (ICU)	95 (63.76%)
General wards	54 (36.24%)
Co-morbidities (n, %)	
COVID-19	21 (14.09%)
Neurocognitive disorder	31 (20.81%)
Hypertension	88 (59.06%)
Congestive heart failure	25 (16.78%)
Diabetes	40 (26.85%)
Depression	41 (27.52%)
Anxiety	30 (20.13%)
Malignancy	22 (14.77%)
Morbid obesity	21 (14.09%)
Delirium subtype (n, %)	
Hyperactive	128 (85.91%)
Mixed	21 (14.09%)

All 149 patients experienced a quantitative reduction in acute sedative use after initiating guanfacine. The average initial dose of guanfacine was 1.82 ± 0.55mg. The mean maximum dose was calculated as 2.61 ± 1.08mg. The average time to a 25% reduction in acute sedative use was 71.59 ± 38.88 hours (Table 2). The choice of rescue sedative agent included: haloperidol (n=88, 59.06%), quetiapine (n=25, 16.78%), lorazepam (n=17, 11.41%), olanzapine (n=12, 8.05%), risperidone (n=4, 2.68%), and chlorpromazine (n=3, 2.01%). The average doses of each rescue agent (within 24 hours) used prior to guanfacine were as follows: haloperidol 12.3 ± 5.8mg, quetiapine 104.7 ± 53.5mg, lorazepam 4.2 ± 1.9mg, olanzapine 9.1 ± 4.6mg, risperidone 2.8 ± 2.1mg, and chlorpromazine 91.8 ± 33.5mg. In a subset of 93 patients initially receiving PT/OT and no longer participating due to delirium-related behavioral agitation, 69 (74.19%) had a documented renewal of services within four days of guanfacine initiation, thus allowing these patients to resume active participation in PT/OT. The mean days without PT/OT prior to this was 4.67 ± 2.08.

**Table 2:** Guanfacine dosing and concomitant acute sedative use.

Intervention	Result
Initial dose of guanfacine (mean ± SD, mg)	1.82 ± 0.55
Maximum dose of guanfacine (mean ± SD, mg)	2.61 ± 1.08
Acute sedative agent (n, %)	
Haloperidol	88 (59.06%)
Quetiapine	25 (16.78%)
Lorazepam	17 (11.41%)
Olanzapine	12 (8.05%)
Risperidone	4 (2.68%)
Chlorpromazine	3 (2.01%)
Acute sedative dosing before guanfacine initiation (mean ± SD, mg)	
Haloperidol	12.3 ± 5.8
Quetiapine	104.7 ± 53.5
Lorazepam	4.2 ± 1.9
Olanzapine	9.1 ± 4.6
Risperidone	2.8 ± 2.1
Chlorpromazine	91.8 ± 33.5

A subset of 112 patients was documented as using opioids for pain relief at the time of guanfacine initiation. Seventy-eight patients (69.64%) experienced a 25% reduction in opioid administration within four days after the initiation of guanfacine (Table 3). The choice of opioid was observed as follows: hydromorphone (intravenous, n=10, 8.93%), morphine (n=13, 11.61%), fentanyl (n=14, 12.50%), oxycodone (n=32, 28.57%), hydrocodone/acetaminophen (n=22, 19.64%), oxycodone/acetaminophen (n=16, 14.29%), and tramadol (n=5, 4.46%). The mean 24-hour doses of each opioid prior to guanfacine initiation were as follows: hydromorphone 2.5 ± 0.9mg, morphine 15.5 ± 4.7mg, fentanyl 82.2 ± 37.5 mcg/hour, oxycodone 14.3 ± 5.4mg, hydrocodone/acetaminophen 21.6 ± 6.1mg, oxycodone/acetaminophen 17.3 ± 5.7 mg, and tramadol 117.6 ± 37.7mg.

**Table 3:** Opioid use and dosing in hospitalized patients receiving guanfacine (subsample).

Intervention	Results
Opioid for pain relief while hospitalized	112 patients
Opioid agent (n,%)	
Hydromorphone (IV)	10 (8.93%)
Morphine	13 (11.61%)
Fentanyl	14 (12.50%)
Oxycodone	32 (28.57%)
Hydrocodone/acetaminophen	22 (19.64%)
Oxycodone/acetaminophen	16 (14.29%)
Tramadol	5 (4.46%)
Mean dose of opioid before guanfacine initiation (mean ± SD, mg)	
Hydromorphone (IV)	2.4 ± 0.9
Morphine	15.5 ± 4.7
Fentanyl	82.2 ± 37.5
Oxycodone	14.3 ± 5.4
Hydrocodone/acetaminophen	21.6 ± 6.1
Oxycodone/acetaminophen	17.3 ± 5.7
Tramadol	117.6 ± 37.7

Cardiovascular adverse effects were also screened for and recorded in all 149 patients (Table 4). No patients experienced consecutive episodes of hypotension that required a change in their clinical care. Two patients experienced a single episode of consecutive bradycardia (48 beats per minute), leading to their primary team physician discontinuing guanfacine. Both patients were in acute septic shock during hospital admission and guanfacine initiation. They were additionally being prescribed multiple vasopressor agents, given their critical status. No other bradycardia events or side effects (e.g., worsening mental status, somnolence, falls) were documented. Guanfacine was also administered in 33 delirious patients with prolonged QTc, with an average QTc of 533.17 msec. No worsening of QTc prolongation was calculated in any of these patients.

**Table 4:** Cardiovascular adverse effects documented after guanfacine initiation.

Adverse Effect	Result
Hypotension (2 or more consecutive episodes of SBP<90mmHg and/or DBP<60mmHg, requiring clinical intervention)	0 patients
Bradycardia (2 or more consecutive episodes of HR<50 beats per minute requiring clinical intervention)	2 patients (both in acute septic shock)
QTc prolongation (Fridericia criteria ≥ 500 msec)	0 (administered in 33 patients with an average QTc of 533.17msec)

## Discussion

This study demonstrates the safety and potential benefits of prescribing guanfacine for managing behavioral agitation and reducing rescue sedative use in patients with hyperactive or mixed-type delirium. This is the first report from an appreciable patient sample that provides such evidence. In our primary outcome analysis, all 149 recorded patients benefitted from the initiation of guanfacine in terms of delirium-related agitation and acute sedative reduction. For our secondary outcomes, we found that patients receiving PT/OT displayed renewal in their participation, reduced opioid usage after initiation of guanfacine, and experienced minimal to no cardiovascular adverse effects.

Our results suggest that adding guanfacine may mitigate basal levels of delirious agitation and allow for lower doses of acute sedatives (antipsychotics in particular) that are required for appropriate chemical sedation. Haloperidol was the most common agent prescribed in our sample, with an average 24-hour dose of 12.3mg; a 25% reduction of this represents a meaningful decrease in exposure to this medication and the side effect burden that it entails. Moreover, of our 93 patients receiving physical or occupational therapy, most demonstrated a renewed ability to participate within four days of guanfacine initiation. This was after receiving no therapy due to their delirium severity and level of cooperation. Of note, active participation in PT has been conclusively shown to decrease the incidence of developing delirium and increases the rate of recovery from delirium [36-38]. The four days were set due to steady state pharmacokinetics [39] based on the average half-life of guanfacine (17 hours) [40]. Thus, the hypothetical time to steady state was estimated to be 68 to 85 hours. Given our mean time to a reduction in acute sedative use (71.59 hours) and the preponderance of patients displaying improved participation (due to less agitation) in PT/OT within four days, this data lent further credence to our hypothesis. Of interest, these positive outcomes were observed with only a mean initial and maximum dose of 1.82mg and 2.61mg, respectively. This is well within the FDA-recommended maximum of 4mg for attention-deficit/hyperactivity disorder. As antipsychotics are used off-label and often in higher than standard doses (e.g., for schizophrenia) to manage delirium, this may demonstrate another benefit of adding guanfacine. Higher average doses of guanfacine may lead to even more clinically observable benefits in agitation. This would have to be explored formally in dose escalation-designed clinical trials.

Regarding secondary outcomes, the reduction in opioid use was considerable. As most of our sample (n=95) was present in the ICU when diagnosed with delirium and initiated on guanfacine, this is an exciting finding. Opioids are commonly prescribed for acute pain relief, especially in the ICU. It is estimated that 80% of critically ill patients are being administered opioids at any time [41]. Effective pain management is complex, and clinicians continue to search for alternative and supplemental drugs since opioid prescriptions and subsequent adverse effects have exponentially increased in recent decades [42].

Furthermore, opioids have been shown to increase the risk of delirium; thus, a decrease in their use would have the added benefit of limiting the overall deliriogenic medication burden [43-46]. A 25% reduction in opioid administration was observed in 69.64% of our subset of 112 patients receiving opioids while simultaneously receiving guanfacine. This is not surprising given previous studies that have reported on the opioid-sparing analgesic properties of dexmedetomidine [47,48], and thus likely to be true for all alpha-2 agonist agents. The proposed mechanism of action here involves agonism of alpha-2 adrenoreceptors in the locus coeruleus and spinal cord and subsequent potassium channel activation and inhibition of voltage-gated calcium channels [49]. Guanfacine may offer an attractive alternative as it is inexpensive and can be administered orally outside the ICU. For the patients who did not experience a reduction in opioid use, one potential explanation is that guanfacine was dosed based on response to agitation, not pain. Future studies may explore optimizing guanfacine dosing to target norepinephrine-mediated analgesia specifically.

Perhaps the most striking outcome observed in our study was the overall safety profile of guanfacine. It is well-known that dexmedetomidine and clonidine use is limited in delirium by their potent cardiovascular adverse effects [50]. We hypothesized that guanfacine would possess a much lower propensity to cause such adverse events, given its significantly higher alpha-2 receptor selectivity. In our sample, there were zero episodes of consecutive hypotension. Two patients experienced consecutive episodes of bradycardia that led to the discontinuation of guanfacine by their primary team physicians. Notably, both patients were diagnosed with acute septic shock and suffered many medical complications. It is unknown whether guanfacine alone caused their symptomatic bradycardia. Thirty-three patients with significant QT prolongation (mean 533.17 msec) benefitted from the initiation of guanfacine for their agitation without worsening their QT intervals. In the initial report submitted by Shire Pharmaceuticals for the FDA approval of guanfacine (Intuniv) for ADHD, a 10 msec increase in QTc was reported in some study participants. Recent follow-up studies have not demonstrated any pro-arrhythmogenic properties [51]. Our data support and advances these safety findings, as our sample consisted of many critically ill patients, not healthy controls. Notably, in these 33 patients with QT prolongation, lorazepam was the common sedative of choice, likely due to concerns regarding antipsychotics and their QT-prolonging capabilities. As our pharmacological inventory for agitation is even more limited in delirious patients with QT prolongation, guanfacine could potentially be especially important in this not-uncommon clinical scenario.

Finally, our study has several limitations that deserve to be mentioned. This was a retrospective descriptive analysis without a control group. We could not select a control due to our sample's heterogeneity and the general wards' inclusion. If we had only conducted a study in the ICU, it would have been more feasible to have a delineated control group. No statistical analyses were conducted as such. As we have demonstrated the safety and possible effectiveness of guanfacine for delirium, future studies should be prospectively designed for clinical trials that include randomization, a control group, and a blind to make direct comparisons. Our sample may suffer selection bias, as this study was conducted at a single, tertiary academic hospital, thus affecting the acuity, severity, and complexity of delirious patients. The average age of our patients was also younger (58.12 years; a range of 19 to 89 years), thus affecting the generalizability of our results to the geriatric population in terms of safety profile. There were no standardized methods for continuously assessing delirium. The outcomes of improved agitation and reduced opioid utilization were not regularly evaluated with any validated objective scales, as seen in a clinical trial. Besides their beneficial sedative and analgesic properties, alpha-2 agonists have also reportedly been helpful for improved sleep architecture in the setting of delirium [52]. We were unable to study this, and thus future endeavors should likely focus on this parameter.

## Conclusions

Based on our retrospective review, we conclude that guanfacine is a safe and well-tolerated medication for patients with hyperactive and mixed-type delirium both in the ICU and medical wards. Even in medically and critically ill patients, cardiovascular adverse events were rare with guanfacine. Patients treated with guanfacine experienced decreased requirements for using sedative agents to manage delirium-associated behavioral agitation. Additionally, patients treated with guanfacine reduced their use of opioid medications and were better able to participate in physical and occupational therapy. Future studies with prospective, randomized, placebo-controlled designs are warranted to evaluate this promising intervention for delirium further.

## References

[1] Maldonado JR (2017). Acute brain failure: pathophysiology, diagnosis, management, and sequelae of delirium. Crit Care Clin.

[2] Leslie DL, Inouye SK (2011). The importance of delirium: economic and societal costs. J Am Geriatr Soc.

[3] Dubois MJ, Bergeron N, Dumont M, Dial S, Skrobik Y (2001). Delirium in an intensive care unit: a study of risk factors. Intensive Care Med.

[4] Inouye SK, Westendorp RG, Saczynski JS (2014). Delirium in elderly people. Lancet.

[5] Morandi A, Piva S, Ely EW (2017). Worldwide Survey of the "Assessing Pain, Both Spontaneous Awakening and Breathing Trials, Choice of Drugs, Delirium Monitoring/Management, Early Exercise/Mobility, and Family Empowerment" (ABCDEF) Bundle. Crit Care Med.

[6] Herzig SJ, Rothberg MB, Guess JR, Gurwitz JH, Marcantonio ER (2016). Antipsychotic medication utilization in nonpsychiatric hospitalizations. J Hosp Med.

[7] Sanders KM, Murray GB, Cassem NH (1991). High-dose intravenous haloperidol for agitated delirium in a cardiac patient on intra-aortic balloon pump. J Clin Psychopharmacol.

[8] Levenson JL (1995). High-dose intravenous haloperidol for agitated delirium following lung transplantation. Psychosomatics.

[9] Broderick PA, Gibson GE (1989). Dopamine and serotonin in rat striatum during in vivo hypoxic-hypoxia. Metab Brain Dis.

[10] Miller R (1984). Major psychosis and dopamine: controversial features and some suggestions. Psychol Med.

[11] Knell AJ, Davidson AR, Williams R, Kantamaneni BD, Curzon G (1974). Dopamine and serotonin metabolism in hepatic encephalopathy. Br Med J.

[12] Trzepacz PT (2000). Is there a final common neural pathway in delirium? focus on acetylcholine and dopamine. Semin Clin Neuropsychiatry.

[13] Trzepacz PT, Leavitt M, Ciongoli K (1992). An animal model for delirium. Psychosomatics.

[14] Maldonado JR (2018). Delirium pathophysiology: an updated hypothesis of the etiology of acute brain failure. Int J Geriatr Psychiatry.

[15] Burry L, Mehta S, Perreault MM (2018). Antipsychotics for treatment of delirium in hospitalised non-ICU patients. Cochrane Database Syst Rev.

[16] Kim MS, Rhim HC, Park A (2020). Comparative efficacy and acceptability of pharmacological interventions for the treatment and prevention of delirium: a systematic review and network meta-analysis. J Psychiatr Res.

[17] Maldonado JR, Wysong A, van der Starre PJ, Block T, Miller C, Reitz BA (2009). Dexmedetomidine and the reduction of postoperative delirium after cardiac surgery. Psychosomatics.

[18] Hansen N, Rediske AI (2021). The locus coeruleus noradrenaline system in delirium. Front Aging Neurosci.

[19] Chiu KM, Lin TY, Lu CW, Wang SJ (2011). Inhibitory effect of glutamate release from rat cerebrocortical nerve terminals by α2 adrenoceptor agonist dexmedetomidine. Eur J Pharmacol.

[20] Li X, Eisenach JC (2001). alpha2A-adrenoceptor stimulation reduces capsaicin-induced glutamate release from spinal cord synaptosomes. J Pharmacol Exp Ther.

[21] Alexopoulou C, Kondili E, Diamantaki E, Psarologakis C, Kokkini S, Bolaki M, Georgopoulos D (2014). Effects of dexmedetomidine on sleep quality in critically ill patients: a pilot study. Anesthesiology.

[22] Venn RM, Grounds RM (2001). Comparison between dexmedetomidine and propofol for sedation in the intensive care unit: patient and clinician perceptions. Br J Anaesth.

[23] Su X, Meng ZT, Wu XH (2016). Dexmedetomidine for prevention of delirium in elderly patients after non-cardiac surgery: a randomised, double-blind, placebo-controlled trial. Lancet.

[24] Turan A, Duncan A, Leung S (2020). Dexmedetomidine for reduction of atrial fibrillation and delirium after cardiac surgery (DECADE): a randomised placebo-controlled trial. Lancet.

[25] Chen K, Lu Z, Xin YC, Cai Y, Chen Y, Pan SM (2015). Alpha-2 agonists for long-term sedation during mechanical ventilation in critically ill patients. Cochrane Database Syst Rev.

[26] Seedat YK (1985). Clonidine and guanfacine--comparison of their effects on haemodynamics in hypertension. S Afr Med J.

[27] Arnsten AF, Cai JX, Goldman-Rakic PS (1988). The alpha-2 adrenergic agonist guanfacine improves memory in aged monkeys without sedative or hypotensive side effects: evidence for alpha-2 receptor subtypes. J Neurosci.

[28] Gertler R, Brown HC, Mitchell DH, Silvius EN (2001). Dexmedetomidine: a novel sedative-analgesic agent. Proc (Bayl Univ Med Cent).

[29] Ernsberger P, Giuliano R, Willette RN, Reis DJ (1990). Role of imidazole receptors in the vasodepressor response to clonidine analogs in the rostral ventrolateral medulla. J Pharmacol Exp Ther.

[30] Jiang S, Czuma R, Cohen-Oram A, Hartney K, Stern TA (2021). Guanfacine for hyperactive delirium: a case series. J Acad Consult Liaison Psychiatry.

[31] Jiang S, Hernandez M, Burke H (2022). A retrospective analysis of guanfacine for the pharmacological management of delirium. Acad Consult Liaison Psychiatry.

[32] Regier DA, Kuhl EA, Kupfer DJ (2013). The DSM-5: classification and criteria changes. World Psychiatry.

[33] Liptzin B, Levkoff SE, Cleary PD, Pilgrim DM, Reilly CH, Albert M, Wetle TT (1991). An empirical study of diagnostic criteria for delirium. Am J Psychiatry.

[34] (2022). International Classification of Diseases, Tenth Revision, Clinical Modification (ICD-10-CM). https://www.cdc.gov/nchs/icd/icd-10-cm.htm.

[35] Fridericia LS (1920). Die Systolendauer im Elektrokardiogramm bei normalen Menschen und bei Herzkranken [German]. Acta Medica Scandinavica.

[36] Needham DM, Korupolu R, Zanni JM (2010). Early physical medicine and rehabilitation for patients with acute respiratory failure: a quality improvement project. Arch Phys Med Rehabil.

[37] Schweickert WD, Pohlman MC, Pohlman AS (2009). Early physical and occupational therapy in mechanically ventilated, critically ill patients: a randomised controlled trial. Lancet.

[38] Wassenaar A, Rood P, Schoonhoven L, Teerenstra S, Zegers M, Pickkers P, van den Boogaard M (2017). The impact of nUrsiNg DEliRium Preventive INnterventions in the Intensive Care Unit (UNDERPIN-ICU): a study protocol for a multi-centre, stepped wedge randomized controlled trial. Int J Nurs Stud.

[39] Levy RH, Bauer LA (1986). Basic pharmacokinetics. Ther Drug Monit.

[40] Sorkin EM, Heel RC (1986). Guanfacine. a review of its pharmacodynamic and pharmacokinetic properties, and therapeutic efficacy in the treatment of hypertension. Drugs.

[41] Wunsch H, Hill AD, Fu L (2020). New opioid use after invasive mechanical ventilation and hospital discharge. Am J Respir Crit Care Med.

[42] Skrobik Y (2020). Insights into critical care and post-ICU opiate administration. Am J Respir Crit Care Med.

[43] Fong HK, Sands LP, Leung JM (2006). The role of postoperative analgesia in delirium and cognitive decline in elderly patients: a systematic review. Anesth Analg.

[44] Duprey MS, Dijkstra-Kersten SM, Zaal IJ (2021). Opioid use increases the risk of delirium in critically ill adults independently of pain. Am J Respir Crit Care Med.

[45] Pandharipande P, Ely EW (2006). Sedative and analgesic medications: risk factors for delirium and sleep disturbances in the critically ill. Crit Care Clin.

[46] Pisani MA, Murphy TE, Araujo KL, Slattum P, Van Ness PH, Inouye SK (2009). Benzodiazepine and opioid use and the duration of intensive care unit delirium in an older population. Crit Care Med.

[47] Su S, Ren C, Zhang H, Liu Z, Zhang Z (2016). The opioid-sparing effect of perioperative dexmedetomidine plus sufentanil infusion during neurosurgery: a retrospective study. Front Pharmacol.

[48] Zhang B, Wang G, Liu X, Wang TL, Chi P (2017). The opioid-sparing effect of perioperative dexmedetomidine combined with oxycodone infusion during open hepatectomy: a randomized controlled trial. Front Pharmacol.

[49] Owesson CA, Seif I, McLaughlin DP, Stamford JA (2003). Different alpha(2) adrenoceptor subtypes control noradrenaline release and cell firing in the locus coeruleus of wildtype and monoamine oxidase-a knockout mice. Eur J Neurosci.

[50] Ebert TJ, Hall JE, Barney JA, Uhrich TD, Colinco MD (2000). The effects of increasing plasma concentrations of dexmedetomidine in humans. Anesthesiology.

[51] Fossa AA, Zhou M, Robinson A, Purkayastha J, Martin P (2014). Use of ECG restitution (beat-to-beat QT-TQ interval analysis) to assess arrhythmogenic risk of QTc prolongation with guanfacine. Ann Noninvasive Electrocardiol.

[52] Skrobik Y, Duprey MS, Hill NS, Devlin JW (2018). Low-dose nocturnal dexmedetomidine prevents ICU delirium. a randomized, placebo-controlled trial. Am J Respir Crit Care Med.

